# Supplementary material for: Empowering refugee voices: Using Nominal Group Technique (NGT) with a diverse refugee Patient Advisory Committee (PAC) to identify health and research priorities in Calgary, Canada
Source: PLoS One. 2025 May 9;20(5):e0323746. doi: 10.1371/journal.pone.0323746 (PMC12064191; doi:10.1371/journal.pone.0323746)
Supplement: S1 Table — (DOCX) [file pone.0323746.s001.docx]

S1 Table. Dates of focus group meetings and their topic of discussion

| Data of PAC Session | Discussion Topic(s) |
| --- | --- |
| January 17^th^, 2023 | **research priorities** for **post-migration period** (up to 2 years). - issues of most importance for refugee health research |
| March 14^th^, 2023 | **health priorities** for **pre-migration/early arrival** period and **post-migration** (up to 2 years) period. |
| May 16^th^, 2023 – Ukrainian Only | **health and research** priorities for the **post-migration** (up to 2 years) period. |
| November 16^th^, 2023 | **health and research** priorities for l**ong term resilient** health system. |
| January 29^th^, 2024 | **research priorities** for the **pre-migration/early arrival** period and prioritized **top 5 across all** health and research priorities. |
