# Supplementary material for: Empowering refugee voices: Using Nominal Group Technique (NGT) with a diverse refugee Patient Advisory Committee (PAC) to identify health and research priorities in Calgary, Canada
Source: PLoS One. 2025 May 9;20(5):e0323746. doi: 10.1371/journal.pone.0323746 (PMC12064191; doi:10.1371/journal.pone.0323746)
Supplement: S2a Table — (DOCX) [file pone.0323746.s002.docx]

S2a Table. Raw rankings of research priorities for pre-migration/early arrival time-period.

| **Priorities** | Total |
| --- | --- |
| What are the most important things that refugees must be aware of when it comes to Canadian healthcare system prior to arrival | **12** |
| How can I navigate/leverage w the education system (ie. Linc classes) to promote healthcare system info, navigation | **12** |
| How can I find out info about medical expenses prior to arrival so I can plan pre-departure? | **12** |
| How can we help make healthcare information more available for refugees post first arrival? | **10** |
| What was your greatest need for your health when you first arrived? | **9** |
| How do I navigate health system? And who can teach me? | **9** |
| Are ppl eligible to receive medication that is important or critical that they use at home in Canada? | **8** |
| Health system orientation pre-departure is critical how | **8** |
| Can Canada introduce health system navigation awareness classes (ie., like mandatory language instruction classes) for health so that all new refugees receive? | **7** |
| How can gov provide a short and complete information that is understandable for post arrival refugees re: vaccinations (routine vs. other) and why important | **7** |
| How do we improve access to pre-departure medical records from home country to Canadian providers | **7** |
| What did you worry about the most before you arrived in Canada? | 5 |
| Upon arrival – how do refugees obtain info about clinics that they can go to? | 5 |
| What to expect when you arrive re: housing, job search, HC, schools, social services and education? | 5 |
| How to deal w pre-existing conditions post arrival? | 5 |
| Possible ways to provide feedback for the IME exams? (i.e., did the clinics submit this information to IRCC?) | 5 |
| Do children or youth receive any assessments re: MH post arrival? Do they receive any access to care, counselling, treatments? | 5 |
| Important health info pre-arrival that is critical for post arrival providers to be safe? How do we make this info available (ie. Woman that is 9months pregnant at departure, is unknown to HC providers post arrival) | 5 |
| IFHP coverage and provincial HC card. How do we improve understanding of both programs and what covers what? | 5 |
| Families are unprepared for the challenges that their children are facing or will face in Canada. How do we support new arrival parents to help and/or support their children (1st gen kids facing issues not common in COO)? | 5 |
| What are the most common or most important health conditions for refugees by global region? | 3 |
| How does finding a job or employment status affect the mental health of newcomers post arrival? | 3 |
| What is the prevalence of communicable and NCDs pre-arrival and how does this differ by global region | 3 |
| How do we improve healthcare providers’ training in Canadian HC system to increase capacity in general | 3 |
| What is the prevalence of MH d/o for children camps vs. no-camps and COO and Region OO? | 2 |
| Question How much to you know about the Canadian HC system? (Question to refugees prior to departure) | 2 |
| How can I communicate w the HC system if I see a violation of conduct by HC employees or institutions? | 2 |
| How can I get health assessment and make me understand it (or results)? | 0 |
| How to IME exam information requirements more clear for refugees pre/post arrival? | 0 |
| Many ppl may be considering coming to Canada as refugees but have health conditions that require HC. How to make info clear whether pre-existing Health conditions would exclude one from coming to Canada? | 0 |
| Why should I receive these vaccinations after arrival? | 0 |
