# Supplementary material for: Empowering refugee voices: Using Nominal Group Technique (NGT) with a diverse refugee Patient Advisory Committee (PAC) to identify health and research priorities in Calgary, Canada
Source: PLoS One. 2025 May 9;20(5):e0323746. doi: 10.1371/journal.pone.0323746 (PMC12064191; doi:10.1371/journal.pone.0323746)
Supplement: S2c Table — (DOCX)\ [file pone.0323746.s004.docx]

S2c Table. Summarized similar priorities into a single sentence with their combined votes.

| One sentence summary | Votes |
| --- | --- |
| 1. How can we make health system information more available or leverage existing programs (i.e. language Classes) to increase health system navigation **post arrival**, including: clinics to go to, vaccinations, insurance coverage, resources, healthcare processes, immigration medical exam, where to start and the importance of each | 55 |
| 2. How can we enhance health system navigation **pre-arrival** as it is critical, which includes: medical expenses orientation, health conditions transition treatments (and whether or not health conditions can affect migration status negatively), and crucial orientation points that refugees should know prior to migration. | 42 |
| 3. How can Canada improve health data transfer in terms of access to medical records from home country and health status and needs to Canadian health system immediately post arrival (ie. Woman that is 9 months pregnant at departure, is unknown to HC providers post arrival). | 17 |
| 4. what were your greatest needs and worries in regards to health prior to coming to Canada? | 14 |
| 5. What is the prevalence of mental health issues for children in camps vs. no-camps and COO and Region OO, and do children or youth receive mental health assessments, care and treatment post arrival and how are parents supported in this regard? | 12 |
