# Supplementary material for: Empowering refugee voices: Using Nominal Group Technique (NGT) with a diverse refugee Patient Advisory Committee (PAC) to identify health and research priorities in Calgary, Canada
Source: PLoS One. 2025 May 9;20(5):e0323746. doi: 10.1371/journal.pone.0323746 (PMC12064191; doi:10.1371/journal.pone.0323746)
Supplement: S2d Table — (DOCX) [file pone.0323746.s005.docx]

S2d Table. Final concise one sentence summary priorities for the research priorities for pre-migration/early arrival time-period (0 – 3 months).

| One sentence summary | Votes |
| --- | --- |
| How to create/improve post-arrival refugee healthcare system navigation? | 55 |
| How to improve pre-arrival refugee healthcare guidance/navigation? | 42 |
| How to ensure pre --> post migration refugee health info continuity sharing? | 17 |
| What are refugees’ key pre-arrival health concerns/worries/needs/expectations? | 14 |
| How to provide refugee children’s mental health & parental supports? | 12 |
