# Supplementary material for: Empowering refugee voices: Using Nominal Group Technique (NGT) with a diverse refugee Patient Advisory Committee (PAC) to identify health and research priorities in Calgary, Canada
Source: PLoS One. 2025 May 9;20(5):e0323746. doi: 10.1371/journal.pone.0323746 (PMC12064191; doi:10.1371/journal.pone.0323746)
Supplement: S3 Study protocol — (DOCX) [file pone.0323746.s006.docx]

# Patient Advisory Committee Protocol

*Refugee Health YYC*

### Overview

The Patient Advisory Committee (PAC) is a community-based participatory research initiative of Refugee Health YYC and Mosaic Refugee Health Clinic (MRHC). Utilizing Nominal Group Technique methods, the PAC will centre the lived experiences of the refugee and newcomer community. The input and perspectives from the members of the PAC will guide and inform the priorities and goals of RHYYC and MRHC as a whole.

### Objectives

The objectives of the Patient Advisory Committee are to:

1. Ensure the meaningful collaborative engagement and participation of people with lived experience within the refugee and newcomer community in the work of Refugee Health YYC
2. Provide an opportunity for participants to actively engage in setting health and research priorities, highlight potential barriers, support research activities, and provide input on the goals and direction of RHYYC
3. Ensure that the research activities of RHYYC are guided, informed, and rooted in the needs and input from the refugee community, through active patient engagement
4. Create an opportunity for current and former patients and community members to share their ideas, skills, experiences, and insights to develop the RHYYC program

### Outreach and Recruitment

- MRHC
- Community Partners, Community Engagement
- Self-selection

### Participation/Expectations/Commitment of PAC

1. Participate in scheduled PAC meetings and activities
2. Share feedback, input, and suggestions for improving the research and community engagement activities
3. Actively participate in the development and implementation of knowledge dissemination strategies
4. Utilize Zoom video/conference calling and telephone to meet with team and community members

### Qualifications/Selection Criteria

PAC members will have the following:

1. Lived experience within the refugee, immigrant, and newcomer community. In particular, self-identified members of “top 10 countries of origin”
   1. Syria, Somalia, Sudan, Kenya, Eritrea, Congo, Iraq, Afghanistan, Mexico, Iran (Source: Top 10 Countries of Origin, 2020Q1, MRHC)

Appendix 1:

Top 10 Countries of Origin 2016 – Q1 2020:


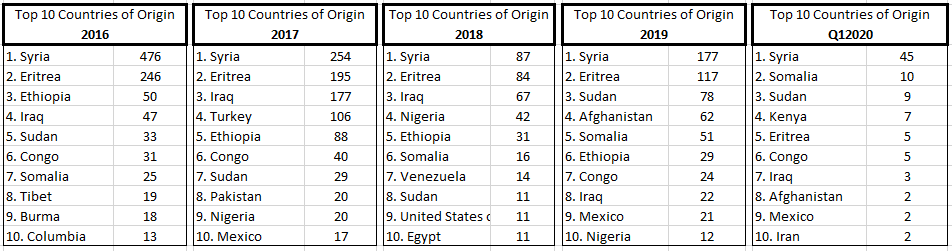


Source: MRHC Data

1. Understanding of the needs and experiences of newcomers to Canada
2. Ability to commit to active participation with the Patient Advisory Committee (~ 1 meeting per month), to help guide the research and development process for RHYYC
3. Demonstrated English language ability
4. Due to COVID-19 precautions, all PAC meetings will be held virtually. Participants must have access to Zoom or a mobile phone to participate in virtual meetings.

### Registration and Screening Process

Interested PAC members will be identified through community partners, community engagement, Mosaic Refugee Health Clinic, and self-selection.

The following questions will be used to register and interested PAC members

1. Name:
2. Are you at least 18 years of age? (Y/N)
3. How would you rate your English language skills in the following areas:
   Reading: Beginner / Moderate / Expert
   Writing: Beginner / Moderate / Expert
   Speaking: Beginner / Moderate / Expert

Interested potential PAC members with at least moderate English proficiency skills in reading and speaking who otherwise fit the study inclusion criteria were invited to join the patient advisory committee (PAC) via either electronic or telephone invitations depending on their stated preference.

### Compensation

All participants of the PAC will be given a gift card in recognition of their time

### Theoretical Framework: Nominal Group Technique (NGT)

What is NGT?

- A process for facilitating small-group discussions and brainstorming sessions, where information is gathered to reach consensus
- In this method, each participant is asked to respond to questions by a moderator. The responses are then prioritized by participants
- This method produces a balanced discussion which allows for disagreement, however NGT is aimed at achieving clarity and consensus among the group
- The end result produces recommendations that reflect the group as a whole, rather than individual members
- Source: [CDC, 2018](https://www.cdc.gov/healthyyouth/evaluation/pdf/brief7.pdf); [American Society for Quality, 2020](https://asq.org/quality-resources/nominal-group-technique#:~:text=Nominal%20group%20technique%20(NGT)%20is,idea%20they%20feel%20is%20best.)

### Materials Needed

- Flipchart (Zoom Whiteboard/Annotations)
- Pens/Marker (Computer Keyboard)

### NGT Process (Modified for use on Zoom) (Adapted from [CDC, 2018](https://www.cdc.gov/healthyyouth/evaluation/pdf/brief7.pdf))

1. Generating Ideas
   1. The Moderator states the problem or question
   2. Silently, each participant considers the topic and develops an idea, solution, or response (5-10 minutes)
2. Recording Ideas
   1. Each participant shares one idea, response, suggestion, opinion, or input
   2. Moderator records each response on flipchart; creates a numbered list
   3. Do not need to repeat if someone has already said the same idea
   4. No discussion or questions permitted; participants are able to freely share responses
   5. Participants are able to “pass”
3. Discussing Ideas
   1. Once all participants have responded, each idea is then discussed: for clarity, to ask questions, agree, or disagree
   2. “Are there any questions or comments group members would like to make about the item?”
4. Voting on Ideas
   1. After discussing each idea, the group votes/ranks/prioritizes (privately) by giving a tally
   2. Moderator develops the criteria used to prioritize ideas
   3. Each person selects the top 5 ideas from the list, writing each on a separate card; most important (5) to least important (1)
   4. The moderator collects all the cards and tallies the points for each idea
   5. The points with the most tallies/highest ratings = most representative of the group

Once priorities were identified across the three resettlement periods, participants convened for a final focus group to determine the overarching priorities in both health and research domains across all three time periods.

1. Summarize and present NGT priorities results
   1. Once priorities were identified across the three resettlement periods, participants convened for a final focus group to determine the overarching priorities in both health and research domains across all three time periods.
   2. Each participant received five stickers to represent their health priorities and five for their research priorities.
   3. Participants were instructed to distribute stickers freely across the previously identified priorities list across three different resettlement periods.
   4. Participants could allocate multiple stickers to a single priority if desired.
   5. Finally, we calculated the total cumulative sticker counts to identify the top five overarching health and research priorities across resettlement time periods.
   6. Following all focus groups, a member-checking step was conducted using an online survey to verify results with PAC participants.
   7. Following all focus groups, a member-checking step was conducted using an online survey to verify results with PAC participants.
   8. Following all focus groups, a member-checking step was conducted using an online survey to verify results with PAC participants.
2. Member checking
   1. Following all focus groups, a member-checking step was conducted using an online survey to verify results with PAC participants.
   2. Findings were shared with participants to verify accuracy and ensure credibility
   3. Consolidated tables of the identified priorities were sent to participants for feedback and confirmation
   4. Participants were asked to provide their opinions on the study’s key findings and their implications for Canadian refugee healthcare.
   5. Member-checking feedback was incorporated into the results.
   6. Finally, participants were invited to review the study manuscript, suggest edits, and co-author if interested.

### Topics for Discussion

1. Health and research priorities pre-migration.
2. Health and research priorities early post-migration.
3. Health and research priorities in the long-term and for a resilient migrant-serving health system.
4. Overarching priorities across all three phases of migration

**References:**

1. Mirambeau, A., Elmi, J., Losby, J., & Gervin, D. (2013). Evaluation Reporting: A Guide to Help Ensure Use of Evaluation Findings. <https://www.cdc.gov/training-development/media/pdfs/2024/04/Evaluation-Reporting-Guide.pdf>
2. American Society for Quality (ASQ). (2025). Nominal group technique (NGT). <https://asq.org/quality-resources/nominal-group-technique>
