# Supplementary material for: Empowering refugee voices: Using Nominal Group Technique (NGT) with a diverse refugee Patient Advisory Committee (PAC) to identify health and research priorities in Calgary, Canada
Source: PLoS One. 2025 May 9;20(5):e0323746. doi: 10.1371/journal.pone.0323746 (PMC12064191; doi:10.1371/journal.pone.0323746)
Supplement: Attachment — Submitted filename: renamed_16eff.docx [file pone.0323746.s009.docx]

PONE-D-24-45096 Response to Reviewers

**Manuscript ID:** PONE-D-24-45096

**Title:** Empowering Refugee Voices: Using Nominal Group Technique with a Diverse Refugee Patient Advisory Committee to Identify Health and Research Priorities in Calgary, Alberta

**Journal:** PLOS ONE

**Dear Associate Editor Dr. Johanna Pruller,**

We greatly appreciate the constructive feedback from the editor and reviewer, which has helped strengthen our manuscript. Below, we provide a point-by-point response addressing all comments. Our revisions are detailed in the manuscript, with corresponding page and line numbers for ease of reference.

Please note that the referenced pages and line numbers are based on the manuscript with tracked changes, which may differ slightly from the clean version.

**Editorial Requirements**

**Comment 1:** Please ensure that your manuscript meets PLOS ONE's style requirements, including those for file naming. The PLOS ONE style templates can be found at

<https://journals.plos.org/plosone/s/file?id=wjVg/PLOSOne_formatting_sample_main_body.pdf> and

[https://journals.plos.org/plosone/s/file?id=ba62/PLOSOne_formatting_sample_title_authors_affiliations.pdf](https://track.editorialmanager.com/CL0/https:%2F%2Fjournals.plos.org%2Fplosone%2Fs%2Ffile%3Fid=ba62%2FPLOSOne_formatting_sample_title_authors_affiliations.pdf/1/010f0194ca7aa216-75be57f5-7492-4ae5-b5f6-5944d4c14b00-000000/GvBzWsUtGehAty3AV9N8XYY2thXIc0WvH6lAFjKVHUk=196)

**Response:** We have made sure to format the manuscript according to PLOS ONE’s style guidelines.

**Comment 2:** We note that the grant information you provided in the ‘Funding Information’ and ‘Financial Disclosure’ sections do not match.

When you resubmit, please ensure that you provide the correct grant numbers for the awards you received for your study in the ‘Funding Information’ section.

**Response:** Thank you. We have corrected this and aligned the Funding Information with the Financial Disclosure section.

**Comment 3:** Clarify the Data Availability Statement to comply with PLOS ONE’s policy.

**Response:** We have revised our Data Availability Statement, ensuring compliance with PLOS ONE’s open data policies. We have uploaded de-identified study data as supplemental information.

**Reviewer 1 Comments**

**Comment 1:** The manuscript states that the study aimed to assess the feasibility and effectiveness of the Nominal Group Technique (NGT) but does not describe how these aspects were evaluated.

**Response:** We appreciate this suggestion and have clarified our approach in the **Methods** section and the **Discussion** section. The added text now reads:

**In the Methods section:** (page 6, lines 129-132)

"We assessed NGT process feasibility among PAC participants using participation rates, session retention, and successful completion of the priority-ranking process. We evaluated NGT process effectiveness depending on whether PAC participants generated actionable health and research priorities and whether participants validated these priorities during a final member checking step.”

**In the Discussion section:** (page 19, lines 398-402)

“The NGT process feasible, as demonstrated by high participation rates, strong session retention, and successful completion of the priority-ranking exercise. It was also effective: participants identified actionable health and research priorities, which were subsequently validated during the member-checking step, confirming their relevance and applicability.”

**Comment 2:**  p5, l. 120: the month should not be abbreviated.

**Response:**  Thank you. We have revised the text and now spell out the month in full as 'January' (page 5, line 122).

**Comment 3:** p5, l. 124: reference [10] refers to an article on cervical cancer screening - please check if this is the appropriate reference at this point and if so, please explain why in your response.

**Response:** Thank you for your comment. We have included reference [10] to cite the 'specialized multidisciplinary refugee clinic' mentioned in that line (page 5, line 125), specifically referring to the Mosaic Refugee Health Clinic in Calgary, Canada. The referenced article discusses this clinic in detail, providing important contextual information.

**Comment 4:** p6, l. 139: Reference [11] refers to an article about COVID-19 outbreaks among migrant workers - the example of meat processing plants - please check if this is the appropriate reference at this point and if so, please explain why in your answer.

**Response:** Thank you for your comment. We included reference [11] to cite one of our studies that provides a more detailed explanation of the role of ‘community scholars’ that we mentioned in that line (page 6, line 142). While the article focuses on COVID-19 outbreaks among migrant workers, it also discusses the involvement of community scholars, making it a relevant reference for this point.

**Comment 5:** p6, l.144: You mention that the study started before February 2022, on p.5, l.120 you state that you conducted the study between January 2023 and May 2024 - please clarify.

**Response:**  Thank you for noting this discrepancy. This was an oversight on our part, and we have made sure to maintain a consistent timeline throughout the study.

The revised text is found on page 6,7, lines 149-152.

“We adapted our protocol to include evacuees from the Canada Ukraine Authorization for Emergency Travel (CUAET) program in March 2022 [13] as Russia’s invasion of Ukraine occurred before the study began in February 2022.”

**Comment 6:**  p6, l.144: please elaborate further on your protocol: is it publicly available or could be provided as supplementary material?

**Response:**  Thank you for this recommendation. We have now included the protocol as supplementary material and have explicitly mentioned this in the manuscript for clarity (page 7, lines 154-155)

**Comment 7:** p8, l. 181-192: Additional information is needed to better understand the NGT format and the respective roles and who is referred to as patient advisor, facilitator and participant. e.g.: What is the difference between patient advisor and participant?

**Response:** Thank you for your feedback. We recognize the potential for confusion and have standardized terminology throughout the manuscript. 'Patient advisor' and 'participant' refer to the same role, and we now exclusively use 'participant(s)' when referring to Patient Advisory Committee (PAC) members.

The facilitator, on the other hand, is any of the study team members and co-authors who guided the sessions and conducted data collection. The different facilitator roles were explained on page 9, line 202-205.

We have added the following sentence on page 9, line 203 to further clarify it: “Study facilitators were study team members and study co-authors.”

**Comment 8:**  p10, l. 233-238: please provide further information on the member verification step to enable the reader to better understand who is considered a 'member'.

**Response:** Thank you for your feedback. To enhance clarity, we have added an explanation on p.10, l.241–245.

“In the member-checking process researchers share findings with participants to verify their accuracy and ensure credibility. In keeping with participatory research principles, it also invites participants to actively engage in the analytic process, thus enhancing the study finding’s accuracy, validity and acceptability [17].”

**Comment 9:**  p10, pg. 242: Who is referred to as a reviewer?

**Response:** Thank you for your comment. In this context, 'reviewer' refers to one of our co-authors, specifically the first author. We have revised the wording in the manuscript to clarify this and explicitly state that the first author performed this role (page 11, line 253-255).

“The first author conducted a thematic analysis of quotes taken verbatim from either field notes or feedback obtained during the member checking step. The analysis was subsequently reviewed and finalized by two other co-authors.”

**Comment 10:**  Please elaborate further on data availability, as the PLOS ONE guidelines make clear: “Stating ‘data available on request from the author’ is not sufficient. If your data are only available upon request, select ‘No’ for the first question and explain your exceptional situation in the text box.”

**Response:** In keeping with the PLOS ONE guidelines, we have now uploaded de-identified study data as supplemental material, ensuring it is accessible to readers. We have updated the manuscript to reflect this, and the data availability statement has been revised accordingly.

**Comment 11:**  The title or at least the abstract should indicate the geographical entity in which the study was conducted.

**Response:** We have revised the title to:

"Empowering refugee voices: Using Nominal Group Technique (NGT) with a diverse refugee Patient Advisory Committee (PAC) to identify health and research priorities in Calgary, Canada”

We have also updated the abstract to clarify the study setting (Page 2, Line 42).

**Comment 12:** p1, l. 5: the affiliation of the first author should be clarified, a is not listed in l.9-22

**Response:**  Apologies for the oversight. We have corrected the first author's affiliation by changing 'a' to '1' on p.1, l.5, and ensuring the affiliation is properly listed in lines 9-24. Additionally, we have added an additional affiliation to reflect the author's current roles.

**Comment 13:**  p12, table 1: the abbreviation IQR needs to be explained

**Response:**  We have added a dagger symbol and defined 'IQR' as 'Interquartile Range' in the footnotes under Table 1 on p.14, line 277 for clarity.

**Comment 14:**  p16, l.321-322: the citation needs explanation/context

**Response:**  We have added an explanation to provide context for the citation on p. 17, lines 342-345, clarifying its relevance to the results.

**Comment 15:**  p16, l.324-325: if this is a (sub)heading, it should not include the reference to figure 3

**Response:**  We have removed the reference to Figure 3 from the subheading and added it to the paragraph below (p.17, l.347-350) for clarity and consistency.

**Comment 16:**  please check that all references comply with the reference style

**Response:**  We have reviewed all references to ensure accuracy and compliance with the journal's style guidelines. We used Zotero as our reference manager and adhered to the Vancouver referencing style as required.

We greatly appreciate the reviewer’s thoughtful feedback and believe these revisions have strengthened the manuscript. We look forward to your further evaluation and are happy to provide additional clarifications if needed.

**Sincerely,**

Gabriel Fabreau MD, MPH, FRCPC (Corresponding Author)
Associate Professor
General Internal Medicine
O’Brien Institute for Public Health
Departments of Medicine and Community Health Sciences
University of Calgary | Cumming School of Medicine

On behalf of all co-authors
